# Association between NoSAS score and cardiovascular disease in patients with obstructive sleep apnea

**DOI:** 10.3389/fcvm.2025.1572055

**Published:** 2025-08-13

**Authors:** Riken Chen, Jie Quan, Qiujing Tang, Zhaojun Chen, Zhongxing Hu, Yitian Yang, Wenxi Li, Yihuan Su, Huizhao Liao, Tingting Sun, Qinghua Chen, Yuli Cai, Weilong Ye, Junfen Cheng, Wenliang Chen, Weimin Yao, Enlin Ye

**Affiliations:** ^1^ Department of Respiratory and Critical Care Medicine, The Second Affiliated Hospital of Guangdong Medical University, Zhanjiang, Guangdong, China; ^2^ Neurology Department, The Second Affiliated Hospital of Guangdong Medical University, Zhanjiang, Guangdong, China; ^3^ Central Hospital of Guangdong Provincial Nongken, Zhanjiang, Guangdong, China; ^4^ The First Clinical School of Medicine, Zhengzhou University, Zhengzhou, Henan, China; ^5^ State Key Laboratory of Respiratory Disease, National Clinical Research Center for Respiratory Disease, Guangzhou Institute of Respiratory Health, The First Affiliated Hospital of Guangzhou Medical University, Guangzhou Medical University, Guangzhou, Guangdong, China; ^6^ Translational Medicine Center, Medical Interdisciplinary Science Research Center of Western Guangdong, The Second Affiliated Hospital of Guangdong Medical University, Zhanjiang, Guangdong, China

**Keywords:** obstructive sleep apnea (OSA), NoSAS questionnaire, morbidity, cardiovascular disease, sleep-related respiratory disorder

## Abstract

**Objective:**

Obstructive sleep apnea (OSA) is a common sleep-related respiratory disorder, yet many cases remain undiagnosed. Given the significant association between OSA and various adverse health outcomes, including cardiovascular complications, early identification and intervention are essential. The NoSAS score effectively screens individuals at high risk of OSA, thereby aiding in early detection.

**Material and methods:**

Data were collected from the Sleep Medicine Center at the First Affiliated Hospital of Guangzhou Medical University and the Sleep Research Institute at the Second Affiliated Hospital of Guangdong Medical University. Participants with a NoSAS score ≥8 were classified as high-risk, while those with scores <3 were classified as low-risk. Logistic regression was used to analyze the association between high-risk classification and cardiovascular disease morbidity.

**Result:**

A total of 2,164 participants with complete NoSAS score data were analyzed. In the high-risk group of 1,248 participants, cardiovascular disease incidence was 7.29%. In the adjusted model, the NoSAS high-risk group demonstrated a 2.2-fold increased risk of cardiovascular disease compared to the low-risk group (OR: 2.22, 95% CI: 1.17–4.18; *p* = 0.006). Age-stratified analysis indicated a significant association between NoSAS risk classification and cardiovascular disease in participants aged ≥60.

**Conclusion:**

In conclusion, the NoSAS high-risk group exhibited a higher burden of cardiovascular disease morbidity and served as an independent predictor of this condition.

## Introduction

Obstructive sleep apnea (OSA) is a common clinical condition characterized by repeated narrowing or collapse of the throat during sleep, leading to apneic events (1). The mechanisms underlying upper respiratory collapse in OSA are not fully understood; however, contributing factors may include obesity, craniofacial anomalies, altered upper respiratory muscle function, pharyngeal neuropathy, and fluid shifts to the neck (1). This chronic sleep disturbance results in daytime sleepiness and fatigue, impairing patients' functional capacity and quality of life. OSA is also associated with hypertension, myocardial infarction, diabetes, cerebrovascular disease, long-term cognitive impairment, and increased mortality (2, 3). Over the past two decades, the global prevalence of OSA has risen, primarily due to the obesity epidemic, an aging population, and advancements in diagnostic technology (4, 5). However, due to the episodic breathing pauses and reduced ventilation during sleep, many individuals with OSA remain undiagnosed and unaware of their condition (5). Given the serious adverse consequences of untreated OSA, timely diagnosis and treatment are essential. The diagnostic gold standard for OSA is nighttime polysomnography (PSG); however, it is time-consuming, labor-intensive, and costly (6). This underscores the urgent need for a practical, reliable method to identify high-risk OSA patients. To address this need, various screening tests have been developed to identify high-risk patients (7, 8).

The NoSAS scoring tool is a practical and effective method for identifying individuals at risk of OSA and has recently been proposed as a screening tool for this condition (9, 10). In two distinct racial cohorts, the negative predictive value (NPV) of the NoSAS tool was 90% and 98%, respectively; thus, it effectively identifies at-risk individuals while excluding those not at risk (9). This study aimed to stratify participants into low-risk and high-risk groups using the NoSAS score and to evaluate association between NoSAS risk stratification and cardiovascular disease morbidity.

## Material and methods

### Data source and participants

Participants were recruited from the Sleep Medicine Center at the First Affiliated Hospital of Guangzhou Medical University and the Sleep Research Institute at the Second Affiliated Hospital of Guangdong Medical University. This study was conducted from September 1, 2016, to October 31, 2020. Ethical approval was obtained from the Ethics Committees of the First Affiliated Hospital of Guangzhou Medical University (Ethics no. 2022183) and the Second Affiliated Hospital of Guangdong Medical University (Ethics no. PJKT2024-050). Informed consent was obtained from all participants.

Inclusion criteria required participants to meet the following four conditions: (1) age 18 or older; (2) total sleep time of more than 4 h; (3) capacity for autonomous behavior and conscious awareness; and (4) ability to complete the questionnaire. Exclusion criteria included any participant meeting the following conditions: (1) history of mental illness or psychological disorder; (2) epilepsy or brain tumors; (3) long-term or ongoing use of sedatives or sleeping pills; (4) severe organ failure preventing test completion; (5) prior diagnosis of obstructive sleep apnea hypopnea syndrome (OSA); (6) incomplete questionnaire responses; (7) total sleep time under 4 h; and (8) central or mixed-type sleep apnea.

Cardiovascular disease morbidity was defined as the combined prevalence of coronary heart disease, heart failure and stroke. The diagnosis of CVD was made by an expert cardiologist based on medical history and imaging results.

### NoSAS score

The primary covariates in this study included sex, age, BMI, neck circumference, systolic blood pressure, diastolic blood pressure, smoking status, alcohol consumption, and diabetes status. The NoSAS score, ranging from 0 to 17, assigns points as follows: 4 points for neck circumference greater than 40 cm; 3 points for a BMI between 25 kg/m^2^ and less than 30 kg/m^2^, or 5 points for a BMI of 30 kg/m^2^ or higher; 2 points for snoring; 4 points for age over 55; and 2 points for being male. In this study, participants with scores of 8 or higher were classified as high-risk, while those scoring below 8 were classified as low-risk.

### Statistical analysis

Continuous variables with a normal distribution were presented as mean [standard deviation (SD)], and an independent-samples *t*-test was conducted to assess differences between groups. Categorical variables were expressed as percentages, and a chi-square test was used to evaluate group differences. Multivariable analyses were conducted to adjust for variables that showed statistical significance in unadjusted analyses. Stratified analyses were conducted based on coronary heart disease, heart failure, and stroke. Additionally, stratified analyses were conducted based on sex, age, and ESS score[An ESS score above 9 indicates excessive daytime sleepiness, multiple ROC curve analyses show that a score of 9 achieves the best balance between sensitivity and specificity (11)]. Multicollinearity was evaluated by calculating variance inflation factors (VIFs) for all independent variables. A VIF <5 was considered acceptable, confirming no significant collinearity. All tests were two-tailed, with statistical significance set at *p* < 0.05. Statistical analyses were conducted using IBM SPSS Statistics for Windows, version 25.0 (IBM Corp., Armonk, NY, USA).

## Result

### Clinical characteristics of high-risk group vs. low-risk groups

A total of 2,164 participants with complete NoSAS questionnaire data were analyzed (Table 1). The low-risk group included 916 participants, with a cardiovascular disease incidence of 1.42%, whereas the high-risk group included 1,248 participants with an incidence of 7.29%. Baseline demographic characteristics for the low-risk and high-risk groups are summarized in Table 1. As expected from the NoSAS scoring criteria, the high-risk group had a higher proportion of men and demonstrated higher mean values for BMI, neck circumference, systolic blood pressure, and diastolic blood pressure compared to the low-risk group.

**Table 1:** Baseline characteristics.

Variables	Total *n* = 2,164	NoSAS low-risk group *n* = 916	NoSAS high-risk group *n* = 1,248	*P*
Prevalence, (%)	104 (4.81)	13 (1.42)	91 (7.29)	
Age (years), $\left(\bar{x}\pm s\right)$	47.68 ± 13.89	43.2 ± 11.83	50.97 ± 14.36	<0.001
Sex (%)				<0.001
Female	479 (22.1)	337 (36.8)	142 (11.4)	
Male	1,685 (77.9)	579 (63.2)	1,106 (88.6)	
BMI, $\left(\bar{x}\pm s\right)$	26.44 ± 4.07	24.33 ± 3.30	27.99 ± 3.89	<0.001
Neck circumference, $\left(\bar{x}\pm s\right)$	38.36 ± 3.93	35.65 ± 2.96	40.34 ± 3.32	<0.001
Systolic blood pressure, $\left(\bar{x}\pm s\right)$	134.95 ± 18.50	128.16 ± 17.41	139.92 ± 187.68	<0.001
Diastolic blood pressure, $\left(\bar{x}\pm s\right)$	83.12 ± 12.31	80.30 ± 11.86	85.19 ± 12.23	<0.001
Smoking (%)				<0.001
Yes	1,318 (60.9)	656 (71.6)	662 (53.0)	
No	846 (39.1)	260 (28.4)	586 (47.0)	
Alcohol drinking (%)				<0.001
Yes	1,614 (74.6)	729 (79.6)	885 (70.9)	
No	550 (25.4)	187 (20.4)	363 (29.1)	
Diabetes (%)				<0.001
Yes	1,997 (92.3)	885 (96.6)	1,112 (89.1)	
No	167 (7.7)	31 (3.4)	136 (10.9)	

### Association of NoSAS risk stratification with cardiovascular disease morbidity

In the unadjusted analysis, factors associated with cardiovascular disease morbidity included age, systolic blood pressure, smoking, and diabetes (Table 2). Additionally, individuals in the high-risk group, compared to those in the low-risk group, demonstrated a significant association with cardiovascular disease morbidity. After adjusting for significant variables from the unadjusted analysis, NoSAS risk stratification remained a significant predictor of increased cardiovascular disease morbidity. The high-risk group demonstrated a 2.2-fold increase in risk compared to the low-risk group (OR: 2.22, 95% CI: 1.17–4.18; *p* = 0.006).

**Table 2:** Risk of cardiovascular disease among all study participants.

Variables	Unadjusted model			Adjusted model		
	OR	95%CI	*P*	OR	95%CI	*P*
Age	1.09	(1.07, 1.11)	<0.001	1.08	(1.06, 1.10)	<0.001
Sex, male	1.00	(0.62, 1.61)	0.996			
Neck circumference	1.01	(0.96, 1.06)	0.772			
BMI	0.99	(0.94, 1.04)	0.605			
Systolic blood pressure	1.02	(1.01, 1.03)	<0.001	1.00	(0.99, 1.01)	0.851
Diastolic blood pressure	0.99	(0.98, 1.01)	0.384			
Smoking	1.66	(1.12, 2.46)	0.012	1.70	(1.11, 2.61)	0.014
Alcohol drinking	0.98	(0.62, 1.54)	0.920			
Diabetes	5.69	(3.60, 9.00)	<0.001	3.62	(2.22, 5.91)	<0.001
NoSAS group						
Low-risk	1.00			1.00		
High-risk	5.46	(3.04, 9.83)	<0.001	2.22	(1.17, 4.18)	0.006

Individuals in the high-risk group, compared to those in the low-risk group, demonstrated a significant association with coronary heart disease morbidity. The high-risk group demonstrated a 1.1-fold increase in risk compared to the low-risk group (OR: 1.11, 95% CI: 1.01–1.22; *p* = 0.034) (Table 2A). However, the adjusted analysis did not show a significant association between NoSAS risk stratification and heart failure and stroke morbidity (Tables 2B, 2C).

**Table 2A:** Risk of coronary heart disease among all study participants.

Variables	Unadjusted model			Adjusted model		
	OR	95%CI	*P*	OR	95%CI	*P*
Age	1.08	(1.06, 1.11)	<0.001	1.06	(1.04, 1.10)	<0.001
Sex, male	1.05	(0.60, 1.60)	0.990			
Neck circumference	1.00	(0.95, 1.05)	0.774			
BMI	0.96	(0.90, 1.04)	0.606			
Systolic blood pressure	1.06	(1.00, 1.08)	<0.001	1.00	(0.99, 1.01)	0.850
Diastolic blood pressure	0.99	(0.95, 1.02)	0.380			
Smoking	1.68	(1.22, 2.46)	0.012	1.75	(1.10, 2.64)	0.016
Alcohol drinking	0.98	(0.60, 1.54)	0.924			
Diabetes	5.72	(3.60, 9.00)	<0.001	3.60	(2.20, 5.92)	<0.001
NoSAS group						
Low-risk	1.00			1.00		
High-risk	2.35	(1.86, 5.44)	0.008	1.11	(1.01 1.22)	0.034

**Table 2B:** Risk of heart failure among all study participants.

Variables	Unadjusted model			Adjusted model		
	OR	95%CI	*P*	OR	95%CI	*P*
Age	1.06	(1.04, 1.11)	<0.001	1.08	(1.04, 1.10)	<0.001
Sex, male	1.00	(0.58, 1.60)	0.994			
Neck circumference	1.02	(0.94, 1.08)	0.775			
BMI	0.99	(0.92, 1.05)	0.604			
Systolic blood pressure	1.02	(0.98, 1.03)	0.748			
Diastolic blood pressure	0.99	(0.94, 1.04)	0.382			
Smoking	1.68	(1.20, 2.48)	0.018	1.74	(1.09, 2.69)	0.016
Alcohol drinking	0.98	(0.62, 1.54)	0.920			
Diabetes	5.70	(3.56, 8.34)	<0.001	3.60	(2.24, 5.90)	<0.001
NoSAS group						
Low-risk	1.00			1.00		
High-risk	1.70	(1.22, 3.04)	0.023	1.09	(0.99,1.18)	0.027

**Table 2C:** Risk of stroke among all study participants.

Variables	Unadjusted model			Adjusted model		
	OR	95%CI	*P*	OR	95%CI	*P*
Age	1.07	(1.06, 1.12)	<0.001	1.08	(1.05, 1.12)	<0.001
Sex, male	1.00	(0.58, 1.64)	0.995			
Neck circumference	1.00	(0.93, 1.09)	0.770			
BMI	0.99	(0.92, 1.06)	0.604			
Systolic blood pressure	1.04	(1.01, 1.06)	<0.001			
Diastolic blood pressure	0.98	(0.80, 1.20)	0.835			
Smoking	1.27	(1.08, 1.48)	0.003	1.42	(1.04, 1.94)	0.028
Alcohol drinking	0.98	(0.80, 1.20)	0.835			
Diabetes	1.58	(1.22, 2.05)	<0.001	1.59	(1.23, 2.05)	<0.001
NoSAS group						
Low-risk	1.00			1.00		
High-risk	1.07	(0.97, 1.18)	0.159	1.06	(0.96, 1.16)	0.257

### NoSAS risk stratification and cardiovascular disease morbidity stratified by sex

In both men (Table 3) and women (Table 4), the NoSAS high-risk group exhibited an association with cardiovascular disease morbidity; however, this association did not reach statistical significance.

**Table 3:** Risk of cardiovascular disease morbidity in males.

Variables	Unadjusted model			Adjusted model		
	OR	95%CI	*P*	OR	95%CI	*P*
Age	1.07	(1.03, 1.11)	<0.001	1.05	(1.00, 1.10)	0.038
Neck circumference	1.06	(0.94, 1.20)	0.342			
BMI	0.96	(0.90, 1.03)	0.275			
Systolic blood pressure	1.03	(1.01, 1.05)	0.012	1.00	(0.98, 1.03)	0.723
Diastolic blood pressure	1.01	(0.97, 1.04)	0.779			
Smoking	1.11	(0.14, 8.67)	0.924			
Alcohol drinking	1.02	(0.60, 1.64)	0.967			
Diabetes	7.57	(2.98, 19.28)	<0.001	5.29	(1.96, 14.30)	0.001
NoSAS group						
Low-risk	1.00			1.00		
High-risk	5.99	(2.41, 14.90)	<0.001	2.67	(0.92, 7.71)	0.072

**Table 4:** Risk of cardiovascular disease morbidity in females.

Variables	Unadjusted model			Adjusted model		
	OR	95%CI	*P*	OR	95%CI	*P*
Age	1.09	(1.07, 1.11)	<0.001	1.09	(1.06, 1.11)	<0.001
Neck circumference	1.00	(0.93, 1.06)	0.891			
BMI	0.97	(0.91, 1.03)	0.274			
Systolic blood pressure	1.02	(1.00, 1.03)	0.010	1.00	(0.99, 1.01)	0.975
Diastolic blood pressure	0.99	(0.97, 1.01)	0.252			
Smoking	1.92	(1.21, 3.05)	0.006	2.04	(1.24, 3.35)	0.005
Alcohol drinking	1.01	(0.63, 1.63)	0.964			
Diabetes	5.22	(3.08, 8.84)	<0.001	3.14	(1.79, 5.52)	<0.001
NoSAS group						
Low-risk	1.00			1.00		
High-risk	6.95	(3.01, 16.06)	<0.001	2.34	(0.96, 5.70)	0.061

### NoSAS risk stratification and cardiovascular morbidity stratified by age

In participants aged ≥65 (Table 5), the unadjusted analysis indicated that both diabetes and NoSAS risk stratification were significantly associated with cardiovascular disease morbidity. After adjustment, NoSAS risk grouping remained a significant predictor for cardiovascular disease morbidity, with the high-risk group exhibiting a 4.4-fold increase in risk compared to the low-risk group (OR: 4.41; 95% CI: 1.34–14.57; *p* = 0.015). For participants under 65, the unadjusted analysis revealed a significantly higher cardiovascular disease morbidity risk in the NoSAS high-risk group compared to the low-risk group. However, the adjusted analysis did not show a significant association between NoSAS risk stratification and cardiovascular disease morbidity (Table 6).

**Table 5:** Risk of cardiovascular disease morbidity among participants aged ≥60.

Variables	Unadjusted model			Adjusted model		
	OR	95%CI	*P*	OR	95%CI	*P*
Sex, male	1.37	(0.73, 2.56)	0.329			
Neck circumference	1.03	(0.96, 1.11)	0.401			
BMI	1.04	(0.97, 1.11)	0.273			
Systolic blood pressure	1.00	(0.96, 1.01)	0.925			
Diastolic blood pressure	0.99	(0.96, 1.01)	0.261			
Smoking	1.60	(0.92, 2.79)	0.098			
Alcohol drinking	1.60	(0.83, 3.10)	0.165			
Diabetes	2.50	(1.26, 4.96)	0.009	2.19	(1.10, 4.36)	0.026
NoSAS group						
Low-risk	1.00			1.00		
High-risk	4.88	(1.49, 16.02)	0.009	4.41	(1.34, 14.57)	0.015

**Table 6:** Risk of cardiovascular disease morbidity among participants aged <60.

Variables	Unadjusted model			Adjusted model		
	OR	95%CI	*P*	OR	95%CI	*P*
Sex, male	1.16	(0.54, 2.52)	0.703			
Neck circumference	1.04	(0.97, 1.12)	0.294			
BMI	0.99	(0.92, 1.06)	0.711			
Systolic blood pressure	1.02	(1.01, 1.04)	0.009	1.01	(0.99, 1.03)	0.156
Diastolic blood pressure	1.01	(0.99, 1.03)	0.375			
Smoking	2.11	(1.16, 3.84)	0.015	1.74	(0.93, 3.23)	0.082
Alcohol drinking	0.96	(0.49, 1.88)	0.915			
Diabetes	9.11	(4.78, 17.34)	<0.001	7.58	(3.92, 14.65)	<0.001
NoSAS group						
Low-risk	1.00			1.00		
High-risk	3.33	(1.64, 6.77)	0.001	2.04	(0.95, 4.37)	0.066

### NoSAS risk stratification and cardiovascular morbidity stratified by ESS score

In participants with ESS scores both above (Table 7) and below 9 (Table 8), the NoSAS high-risk group demonstrated a significant association with cardiovascular disease morbidity.

**Table 7:** Risk of cardiovascular disease morbidity among those whose ESS > 9.

Variables	Unadjusted model			Adjusted model		
	OR	95%CI	*P*	OR	95%CI	*P*
Age	0.85	(0.41, 1.74)	0.653			
Sex, male	1.09	(1.06, 1.12)	<0.001	0.50	(0.23, 1.08)	0.079
Neck circumference	0.98	(0.91, 1.05)	0.551			
BMI	0.95	(0.87, 1.02)	0.154			
Systolic blood pressure	1.02	(1.01, 1.04)	0.004	1.01	(0.99, 1.03)	0.120
Diastolic blood pressure	1.00	(0.98, 1.02)	0.999			
Smoking	1.39	(0.77, 2.52)	0.274			
Alcohol drinking	0.593	(0.28, 1.25)	0.167			
Diabetes	4.58	(2.31, 9.10)	<0.001	3.40	(1.69, 6.86)	0.001
NoSAS group						
Low-risk	1.00			1.00		
High-risk	6.88	(2.45, 19.35)	<0.001	6.16	(2.07, 18.28)	0.001

**Table 8:** Risk of cardiovascular disease morbidity among those whose ESS ≤ 9.

Variables	Unadjusted model			Adjusted model		
	OR	95%CI	*P*	OR	95%CI	*P*
Age	1.12	(0.60, 2.11)	0.718			
Sex, male	1.09	(1.07, 1.11)	<0.001	0.51	(0.24, 1.09)	0.081
Neck circumference	1.03	(0.96, 1.10)	0.371			
BMI	1.02	(0.96, 1.09)	0.577			
Systolic blood pressure	1.02	(1.00, 1.03)	0.034	1.01	(0.99, 1.02)	0.454
Diastolic blood pressure	0.99	(0.96, 1.01)	0.221			
Smoking	1.91	(1.13, 3.24)	0.016	2.02	(1.09, 3.72)	0.025
Alcohol drinking	1.42	(0.79, 2.54)	0.239			
Diabetes	6.89	(3.72, 12.78)	<0.001	5.37	(2.84, 10.16)	<0.001
NoSAS group						
Low-risk	1.00			1.00		
High-risk	4.85	(2.36, 9.97)	<0.001	4.08	(1.86, 8.97)	<0.001

## Discussion

In this study, after adjusting for other variables influencing cardiovascular risk, the NoSAS high-risk group remained significantly associated with coronary artery disease morbidity, especially coronary heart disease. When stratified by age, participants aged ≥60 in the high-risk group exhibited a 2.2-fold increased risk of coronary artery disease morbidity compared to the low-risk group.

A growing body of research indicates a correlation between OSA and coronary artery disease morbidity. A longitudinal study in Finland, with up to 523,372 person-years of follow-up, demonstrates that OSA is an independent risk factor for coronary heart disease (12), significantly increasing the risk of this condition. A study with an average follow-up of 10.1 years found that participants with untreated severe OSA experienced a higher incidence of fatal and non-fatal cardiovascular events than healthy participants (13). The Sleep Heart Health Study (SHHS), a large multicenter study, confirmed a significant relationship between OSA, coronary heart disease, and myocardial infarction, supporting the conclusion that OSA increases coronary heart disease incidence (14).

OSA treatment may mitigate cardiovascular risk. A prospective study showed that OSA treatment in coronary artery disease patients was associated with a reduced incidence of new cardiovascular events and a delayed onset of these events (15). In a study by Marin et al., patients with severe OSA receiving CPAP treatment demonstrated a reduced risk of cardiovascular morbidity (16).

Given that OSA is a recognized independent risk factor for cardiovascular disease (17) and that the NoSAS questionnaire has proven effective for OSA screening (18), using this tool allows for the timely identification of individuals at risk. We recommend that individuals identified as high-risk by the NoSAS score undergo further cardiovascular health examinations to enable early detection and timely, effective treatment measures to reduce cardiovascular disease incidence and mortality.

This study has several limitations that should be addressed. First, as a cross-sectional analysis, this study cannot establish a causal relationship between OSA and cardiovascular disease. Although a strong association between OSA and adverse cardiovascular outcomes is well-recognized, further prospective studies are needed to determine if the NoSAS score independently predicts future cardiovascular outcomes. Second, as this analysis was conducted solely on Chinese participants, the findings may not be generalizable to other ethnic groups. Validating these findings in cohorts representing diverse ethnicities is essential to confirm their generalizability.

## Conclusion

In conclusion, the NoSAS high-risk group exhibited a higher burden of coronary artery disease morbidity, especially coronary heart disease. Given the growing evidence linking OSA to an elevated risk of cardiovascular disease, along with the effectiveness of OSA treatment, it is essential to raise public awareness and allocate resources to strengthen early detection and treatment efforts. In addition, further longitudinal studies are needed to explore the association.

## Data Availability

The raw data supporting the conclusions of this article will be made available by the authors, without undue reservation.
